# CASP9 As a Prognostic Biomarker and Promising Drug Target Plays a Pivotal Role in Inflammatory Breast Cancer

**DOI:** 10.1155/2022/1043445

**Published:** 2022-09-25

**Authors:** Mingdi Zhang, Kejin Wu, Maoli Wang, Fang Bai, Hongliang Chen

**Affiliations:** Department of Breast Surgery, Obstetrics and Gynecology Hospital of Fudan University, Shanghai, China

## Abstract

**Background:**

Inflammatory breast cancer (IBC) is one of the most rare and aggressive subtypes of primary breast cancer (BC). Our study aimed to explore hub genes related to the pathogenesis of IBC, which could be considered as novel molecular biomarkers for IBC diagnosis and prognosis. *Material and Methods*. Two datasets from gene expression omnibus database (GEO) were selected. Enrichment analysis and protein-protein interaction (PPI) network for the DEGs were performed. We analyzed the prognostic values of hub genes in the Kaplan-Meier Plotter. Connectivity Map (CMap) and Comparative Toxicogenomics Database (CTD) was used to find candidate small molecules capable to reverse the gene status of IBC.

**Results:**

157 DEGs were selected in total. We constructed the PPI network with 154 nodes interconnected by 128 interactions. The KEGG pathway analysis indicated that the DEGs were enriched in apoptosis, pathways in cancer and insulin signaling pathway. PTEN, PSMF1, PSMC6, AURKB, FZR1, CASP9, CASP6, CASP8, BAD, AKR7A2, ZNF24, SSX2IP, SIGLEC1, MS4A4A, and VSIG4 were selected as hub genes based on the high degree of connectivity. Six hub genes (PSMC6, AURKB, CASP9, BAD, ZNF24, and SSX2IP) that were significantly associated with the prognosis of breast cancer. The expression of CASP9 protein was associated with prognosis and immune cells infiltration of breast cancer. CASP9- naringenin (NGE) is expected to be the most promising candidate gene-compound interaction for the treatment of IBC.

**Conclusion:**

Taken together, CASP9 can be used as a prognostic biomarker and a novel therapeutic target in IBC.

## 1. Introduction

Inflammatory breast cancer (IBC) is one of the most rare and aggressive subtypes of primary breast cancer because of its high metastatic potential [1–3]. The presence of dermal tumor emboli, although not mandatory for the diagnosis, is a distinguishing pathologic characteristic of IBC [4]. Tumor emboli which are nonadherent cell clusters, spreads rapidly by disseminates continuously, thus accelerates distant metastasis and local recurrence [5]. Despite substantial progresses in multi-modality treatment, the survival status of patients with IBC is still poor [6, 7]. A substantiated understanding of the biology of IBC is required to improve treatment and increase survival.

Caspase (CASP) proteins, encoded by genes of the *CASP* family play an important role in the induction, transduction, and amplification of intracellular apoptotic signals [8]. Among these proteins, the CASP9 protein acts as an initiator caspase of apoptosis in the mitochondrial cell death pathway. The upregulation of CASP9 can markedly induce the apoptosis of diverse cells [9]. Further studies have emphasized the role of CASP9 in translational medicine in cancer therapy [10].

In this study, we performed an integrated analysis of two microarray datasets (GSE17907 and GSE45581) from gene expression omnibus (GEO) database and identified the differentially expressed genes (DEGs) between IBC and non-IBC. Then we conducted pathway enrichment to expound biological function of these DEGs in IBC. The hub genes correlated with the pathogenesis of IBC were evaluated by protein-protein interaction (PPI) module analysis and prognosis analysis. Finally, the Connectivity Map (CMap) database was used to explore small molecules targeted for IBC. Based on the potential biomarkers, we predicted the usefulness of naringenin (NGE) in potentially targeting IBC and identify the correlated gene, CASP9 as the survival associated gene.

## 2. Materials and Methods

### 2.1. Data Resources

We searched microarray datasets relating to IBC in GEO database to explore the DEGs between IBC and non-IBC samples. The profiles of GSE17907 and GSE45581 were downloaded from the GEO database (https://www.ncbi.nlm.nih.gov/geo/). These RNA profiles were provided on GPL570 (Affymetrix Human Genome U133 Plus 2.0 Array) and GPL6480 (Agilent-014850 Whole Human Genome Microarray).

### 2.2. Differentially Expression Analysis

The criteria of DEGs were genes with *P* value <0.05. To screen the significantly expressed gene in intersecting part, Venn diagram webtool (bioinformatics.psb.ugent.be/webtools/Venn/) was used to select the overlapping DEGs.

### 2.3. Function Enrichment Analysis

To further analyze the biological potential of the overlapping DEGs, we analyzed gene ontology (GO) terms based on biological process (BP), molecular function (MF), and cellular component (CC). We also conducted Kyoto encyclopedia of genes and genomes (KEGG) pathway analysis to identify the potential signaling pathways of the overlapping DEGs via database for annotation visualization and integrated discovery (DAVID).

### 2.4. Comprehensive Analysis of PPI Network and Hub Module Identification

The search tool for the retrieval of interacting genes (STRING, https://string-db.org/) database was used to structure the PPI network. Subsequently, the functional modules were screened by the Molecular Complex Detection (MCODE) plug-in. Additionally, the hub genes were identified based on the interaction edges and the high degree of connectivity. Meanwhile, the function of module genes was analyzed by GO and KEGG enrichment. The interaction between genes and degree of connectivity will be clearly shown in the network.

### 2.5. Clinical and Survival Analysis of Hub Genes

To further verify the function of hub genes selected from the bioinformatics analysis results, the value of hub genes on overall survival (OS) were evaluated by Kaplan-Meier Plotter database (kmplot.com/analysis), an online database including gene expression data and clinical data. The patient samples were divided into two cohorts according to the median expression of the gene (high vs. low expression). The Kaplan-Meier Plotter database will help to assess prognostic value of a specific gene.

The PrognoScan online database (https://www.prognoscan.org/) provides a powerful platform for assessing the biological relationships between gene expression and prognostic information in cancer patients. The PrognoScan includes public microarray datasets with clinical annotation of gene expression and prognosis from gene expression omnibus (GEO), ArrayExpress, and individual laboratory websites. The correlation between CASP9 expression and survival in breast cancers was analyzed by the PrognoScan database. Cox *P*-values and hazard ratio (HR) with 95% confidence intervals were calculated automatically according to the mRNA level (high or low).

The breast cancer gene-expression miner v4.6 (bc-GenExMiner v4.6, https://bcgenex.ico.unicancer.fr/) is a breast cancer gene expression mining program. Bc-GenExMiner is employed to examine the relationship of molecular subtypes or gene expression patterns, with disease prognosis. Here, we used bc-GenExMiner to determine the relationship of different clinical features with CASP9 expression.

### 2.6. Prediction of Novel Drugs for IBC

The Connectivity Map (CMap, https://www.broadinstitute.org/cmap/) was used to identify the candidate small molecule drugs based on the gene expression of IBC. The enrichment scores representing the similarity ranged from −1 to +1. A positive connectivity value revealed the possibility of a small molecule inducing the gene signature of IBC cells, while a negative connectivity value indicated that a small molecule could reverse the status of non-IBC cells. Then, the candidate drugs were validated using the Comparative Toxicogenomics Database (CTD), which contains association among chemicals, gene products, and diseases [11]. The gene-compound interaction network was visualized by Cytoscape.

### 2.7. Tumor Immune Estimation Resource

The infiltration level of immune cells in breast cancer was predicted using the TIMER database (https://cistrome.shinyapps.io/timer/) [12] to estimate the relationship between CASP9 expression and the abundance of immune infiltration. The correlation between CASP9 expression and the infiltration level of immune cells, including CD8+ *T* cells, CD4+ *T* cells, *B* cells, macrophages, neutrophils, and dendritic cells were analyzed using the Spearman correlation test.

## 3. Results

### 3.1. Analysis of DEGs in IBC

**Figure 1:**
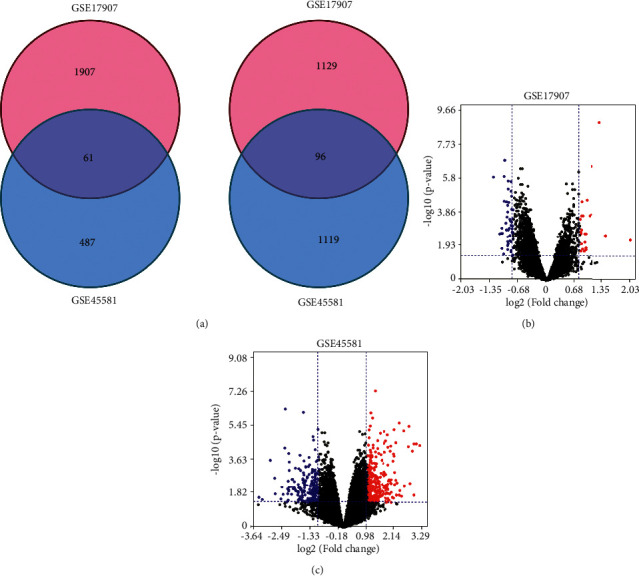
Identification of the DEGs from two IBC microarrays. (a) Venn diagram of 157 overlap DEGs analyzed in two GEO datasets. Volcano plot of gene expression between IBC and non-IBC tissues in GSE45581 (b) and GSE17907 (c). Red dots indicated significantly upregulated genes in IBC; blue dots indicated significantly downregulated genes in IBC; black dots indicated nondifferentially expressed genes. *P* < 0.05 were considered as significant.

Two datasets (GSE45581 and GSE17907) were selected in this study, including 20 IBC samples and 20 non-IBC samples in GSE45581, and 21 IBC specimens and 30 non-IBC specimens in GSE17907.157 overlapping DEGs in total were detected by comprehensive analysis in which 61 genes were significantly upregulated and 96 genes were downregulated (Figure 1(a)). The volcano plot indicated the abnormally expressed genes in two datasets (Figures 1(b) and 1(c)).

### 3.2. Function Analysis of DEGs

**Figure 2:**
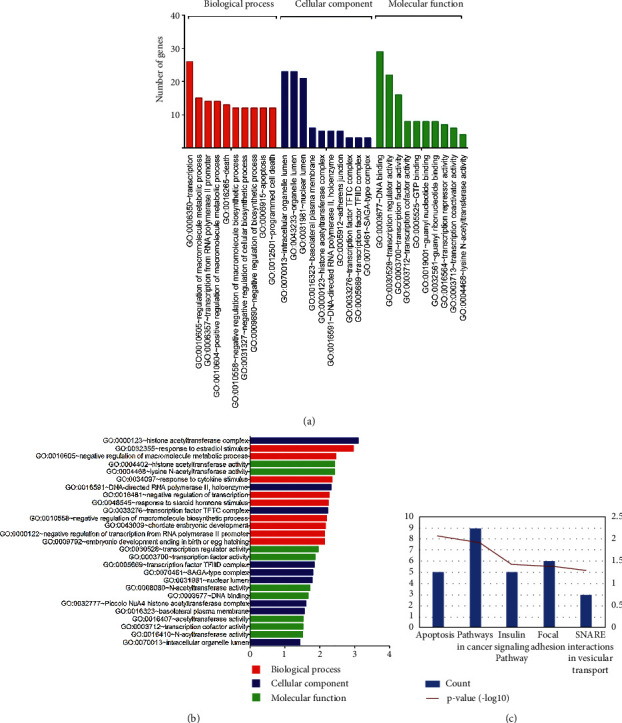
GO and KEGG of DEGs from two microarrays. (a) Sorted by descending order of the number of genes associated with the listed GO ID. (b) Sorted by descending order of −Log10 (*P*-value) for the GO enrichment terms. (c) KEGG pathway of DEGs expressed in inflammatory breast cancer using the DAVID database.

The GO term and KEGG pathway analysis for DEGs were performed to classify the function (Figure 2 and Table 1). GO analysis results showed that the overlap DEGs were significantly enriched in response to estradiol stimulus and transcription in biological processes. Molecular function analysis of the overlap DEGs were obviously enriched in histone acetyltransferase activity and DNA binding. Cell component analysis revealed that the overlap DEGs were enriched in histone acetyltransferase complex and intracellular organelle lumen (Figures 2(a) and 2(a)). In addition, KEGG pathway enrichment analysis indicated that the overlap DEGs were particularly involved in apoptosis, pathways in cancer, and insulin signaling pathway (Figure 2(c)).

### 3.3. Significant Modules Selected from PPI Network and Hub Genes Identification

**Figure 3:**
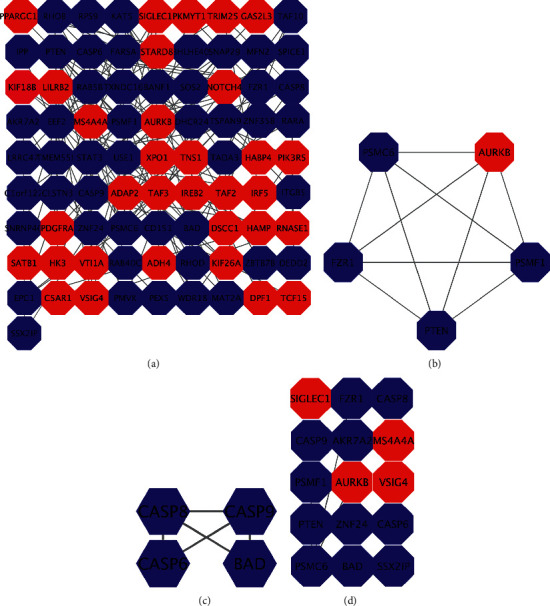
Module screening and functional analysis of the PPI network. (a) Protein-protein interaction network mapping the differentially expressed genes. (b) Module 1 generating from the PPI network. (c) Module 2 generating from the PPI network. (d) Shows fifteen hub genes with a high degree of connectivity. The red nodes represent upregulated DEGs, while the blue nodes represent downregulated DEGs. DEG: differential expression gene; PPI: protein-protein interaction.

We establish the PPI network containing 154 nodes and 128 interactions via the STRING website (Figure 3(a)). The two core modules were selected from the PPI network using MCODE (Figures 3(b) and 3(c)). The biological process of the genes in the two core modules was mostly involved in positive regulation of ubiquitin protein ligase activity and execution phase of apoptosis (Table 2). PTEN, PSMF1, PSMC6, AURKB, FZR1, CASP9, CASP6, CASP8, BAD, AKR7A2, ZNF24, SSX2IP, SIGLEC1, MS4A4A, and VSIG4 with high degree of connectivity were considered as hub genes (Figure 3(d)).

### 3.4. Survival Analysis of Hub Genes in Breast Cancer

**Figure 4:**
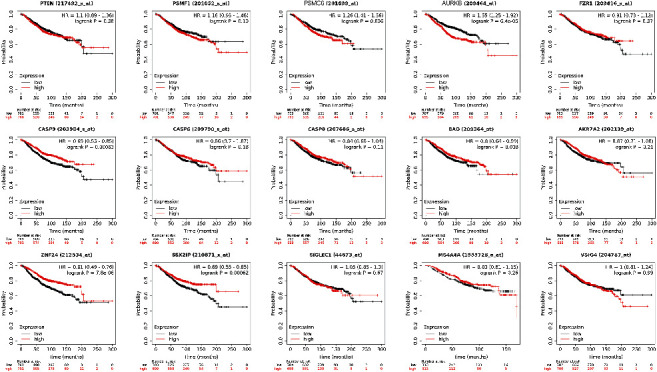
Survival analyses for fifteen hub genes in breast cancer patients. Survival curves show that the expression of PSMC6, AURKB, CASP9, BAD, ZNF24, and SSX2IP was associated with a worse overall survival rate (*P* < 0.05).

The prognostic value of hub genes was evaluated scientifically by Kaplan-Meier-Plotter. As illustrated in Figure 4, we unearthed that the expression of PSMC6 (HR: 1.26 [1.01–1.56], *P*=0.036), AURKB (HR: 1.55 [1.25–1.92], *P*=6.4*e* − 05), CASP9 (HR: 0.69 [0.55–0.85], *P*=0.00063), BAD (HR: 0.8 [0.64–0.99], *P*=0.038), ZNF24 (HR: 0.61 [0.49–0.76], *P*=7.8*e* − 06), and SSX2IP (HR: 0.69[0.55–0.85], *P*=0.00062) were associated with OS in breast cancer patients, whereas PTEN, PSMF1, FZR1, CASP6, CASP8, AKR7A2, SIGLEC1, MS4A4A, and VSIG4 have no significant correlation (*P* > 0.05).

### 3.5. NGE Might be Effective to Treat IBC by Targeting CASP9

All DEGs were imported into the CMap database to identify small molecule drugs between IBC tissues and non-IBC tissues, which may have potential anti-IBC activity. The enrichment scores and *P* value were listed in Table 3. The negative correlation of the above candidate compounds were highly significant, which indicates that these compounds are capable of reversing the gene expression induced by IBC.

**Figure 5:**
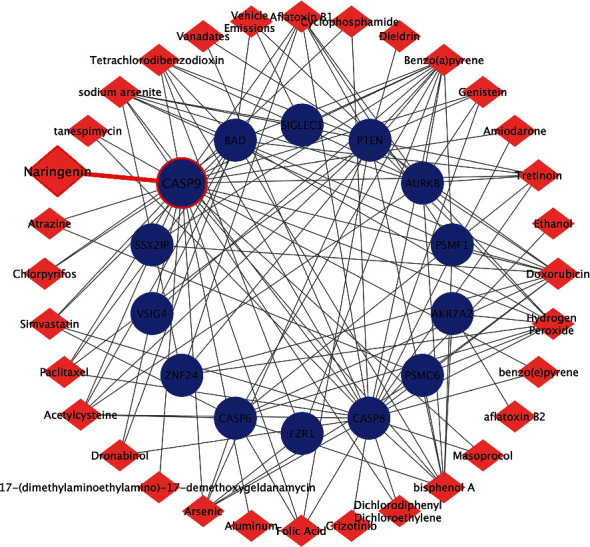
Establishment of gene-compound interaction network. Drug targets of hub genes using the data from CTD and visualized by cytoscape. Red: chemicals; blue: genes.

Also, the CTD was applied to analyze the literature of the candidate compounds and gene (Figure 5). It was found that all the hub genes besides MS4A4A have been studied related to IBC and several compounds have been interacted with IBC-related genes, suggesting that feasibility of screening anti-IBC compounds in our study. Notably, through the gene-compound interaction network, we observed that CASP9-NGE is expected to be the most promising candidate gene-compound interaction for the treatment of IBC (Figure 5).

### 3.6. The Relationship between CASP9 Expression and Clinical Indicators in Breast Cancer Patients

By using the bc-GenExMiner online tool, we compared CASP9 expression among groups of patients, according to different clinical indicators. Estrogen receptor (ER) and progesterone receptor (PR) status were positively associated with CASP9 expression (Table 4), and human epidermal growth factor receptor-2 (HER-2) status were negatively associated withCASP9 expression. Breast cancer patients with wild type P53 showed increased level of CASP9 than those with Mutatedn P53 (Table 4). Besides, we found that CASP9 was strongly elevated in non-basal-like subtype with respect to basal-like subtype; the same pattern of change was also observed in triple-negative breast cancer (TNBC) patients (Table 4). Furthermore, the PrognoScan database showed that overexpression of CASP9 was significantly associated with inferior OS, disease free survival, disease specific survival, distant metastasis free survival, and relapse free survival (Table 5).

### 3.7. CASP9 mRNA Levels Are Associated with Tumor-Infiltrating Immune Cells in Breast Cancer

**Figure 6:**
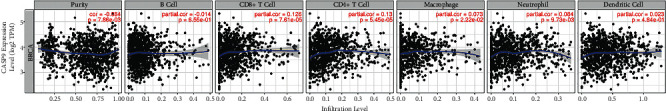
Correlation of CASP9 expression with immune infiltration level in breast cancer. CASP9 expression is negatively correlated with tumor purity and infiltrating level of *B* cells and has significant positive correlations with infiltrating levels of CD8+ *T* cells (*r* = 0.126, *P*=7.61*e* − 05), CD4+ *T* cells (*r* = 0.13, *P*=5.45*e* − 05), macrophages (*r* = 0.073, *P*=2.22*e* − 02), neutrophils (*r* = 0.084, *P*=9.73*e* − 03), and dendritic cells (*r* = 0.023, *P*=4.84*e* − 01) in breast cancer.

In order to explore the relationship between CASP9 expression and immune infiltration in breast cancer, we used the TIMER to further analyze the association between CASP9 expression and immune infiltration levels in breast cancer. We found that CASP9 expression is significantly positively related to the infiltration levels of CD8+ *T* cells (*r* = 0.126, *P*=7.61*e* − 05), CD4+ *T* cells (*r* = 0.13, *P*=5.45*e* − 05), macrophages (*r* = 0.073, *P*=2.22*e* − 02), neutrophils (*r* = 0.084, *P*=9.73*e* − 03), and dendritic cells (*r* = 0.023, *P*=4.84*e* − 01) (Figure 6), indicating that CASP9 promotes immune infiltration in breast cancer.

## 4. Discussion

Inflammatory breast cancer (IBC) is a rare type of breast cancer which accounts for only 2% to 4% of all breast cancer [13, 14]. Despite its low incidence, the burden of IBC morbidity and mortality is significant [15]. Approximately 30% of IBC patients have distant metastases at diagnosis [16]. The absence of precise diagnostic criteria for IBC makes it difficult to investigate the prognosis of IBC.

In our study, an integrative bioinformatics analysis of IBC based on the GEO database was conducted. Totally, 41 IBC samples and 50 non-IBC samples were screened out for the DEGs. We identified 61 upregulated genes and 96 downregulated genes in the two gene expression profiles. These DEGs were important in the progression of IBC and could be potential targets for the IBC treatment. To further understand the function of the DEGs, the functional and pathway enrichment analysis was conducted. The KEGG pathway analysis focused on apoptosis, pathways in cancer, and insulin signaling pathway. The insulin signaling pathway play an important role in the cancer cells growth and progression. Insulin and IGFs have been reported to stimulate renal cell carcinoma cells growth and migration [17]. miR-29a plays crucial roles in decreasing glucose-stimulated insulin secretion, as well as in regulating breast cancer cell proliferation and EMT [18].

To further clarify the interaction between DEGs, we screened fifteen hub genes by constructing the PPI network, among which the expression of PSMC6, AURKB, CASP9, BAD, ZNF24, and SSX2IP was associated with OS in breast cancer patients, which attracted our attention. PSMC6 belongs to PSMC family members [19]. PSMC6 might be candidate biomarkers associated with apoptosis in Melanosis coli and osteoblast [19, 20]. AURKB plays a key role during mitosis [21]. The role of the AURKB expression in cancer prognosis is controversial. AURKB was found to be correlated with the cell proliferation and AURKB expression is an independent prognostic index of breast cancer, especially for triple negative breast cancer (TNBC) [22, 23]. While AURKB expression was reported to be not associated with the survival of breast cancer patients [24]. Recent study demonstrated that high expression of AURKB might induce EMT in breast cancer [25]. Caspase-9 is a key caspase in intrinsic apoptosis pathway. Previous studies have proved that CASP9 protein acts as an initiator caspase of apoptosis in the mitochondrial cell death pathway. Further studies have emphasized the role of CASP9 in translational medicine in cancer therapy [10]. BAD, Bcl-2-associated death promoter, modulates breast cancer cell proliferation and tumor progression by regulating cell cycle progression, sensitizes breast cancer cells to chemotherapy [26, 27]. SSX2 interacting protein (SSX2IP) was proposed to modulate the activity of SSX2 in the testis and malignant cells [28]. Many studies reported that SSX2IP as an acute myeloid leukemia-associated antigen, is a potential immunotherapy target for leukemia [29, 30]. It is also speculated that SSX2IP plays an important role in the development and metastasis of gastric cancer and liver cancer [31]. ZNF24 functioned as a negative regulator of tumor development in breast cancer and gastric cancer [32, 33]. ZNF24 also acted as an oncogene and promoted EMT of prostate cancer cells [34]. However, mechanisms of these genes in IBC still need extensive experimental research to reveal.

As the development of new drugs is a time-consuming and high-risk process, in this case, through gene expression profile technology to find anti-IBC drug targets, the use of drug repositioning technology to explore the novel efficacy of existing drugs, so as to achieve “new uses of old drugs,” which has become an effective measure to improve the input-output ratio of drug development and reduce the risk of failure [35]. In our study, ten candidate small molecular compounds were identified via CMap database. Based on the interaction between candidate small molecular compounds and hub genes, we proposed that CASP9-NGE is expected to be the most promising candidate gene-compound interaction for the treatment of IBC. NGE, natural citrus flavonoids, have a number of functions including antioxidant, anti-inflammatory, anti-ulcer, anti-apoptotic, and anti-carcinogenic activities [36, 37]. Furthermore, NGE have been proved to inhibit tumor cell proliferation and stimulate cell apoptosis, including breast, bladder, and cervical cancers [38]. NGE exerts an anticancer effect on breast cancer by altering the biochemical and antioxidant parameters related to inflammation [39].

The study on bioinformatics analysis of IBC core genes has been performed. A limited number of signaling pathways was clearly evaluated [40–46]. Some of the upregulated genes could offer useful diagnostic or prognostic markers and predict the effect of neoadjuvant therapy [47, 48]. Previous study identified a large number of NF-kappaB target genes and the insulin-like growth factor-signaling pathway, potentially contributing to the aggressive nature of IBC [49, 50]. More importantly, this integrated bioinformatics analysis for IBC have found out the gene-compound interaction, CASP9-NGE, for the treatment of IBC.

## 5. Conclusion

In conclusion, the comprehensive bioinformatics analysis has identified novel genes and important pathways in IBC. We then identified six hub genes (PSMC6, AURKB, CASP9, BAD, ZNF24, and SSX2IP) that were significantly associated with the prognosis of breast cancer. The results above revealed that six hub genes may play a pivotal role in IBC pathogenesis and progression. Additionally, candidate compounds with potential anti-IBC activity were predicted, in which CASP9-NGE is regarded as the most promising gene-compound interaction treating IBC. Our study may provide new insight into the molecular mechanism of IBC progression and also guide the development of anti-IBC treatment target, which are expected to improve the prognosis of IBC.

## Figures and Tables

**Table 1:** Functional analysis of the intersected DEGs.

Category	Term	Count	Fold enrichment	*P* value
GOTERM_BP_FAT	GO: 0006350∼transcription	26	1.455737434	0.0433219
GOTERM_BP_FAT	GO: 0010605∼macromolecule metabolic process	15	2.403980571	0.0034352
GOTERM_BP_FAT	GO: 0006357∼transcription from RNA polymerase II promoter	14	2.26531906	0.0081237
GOTERM_BP_FAT	GO: 0010604∼positive regulation of macromolecule metabolic process	14	1.921688397	0.0280958
GOTERM_BP_FAT	GO: 0016265∼death	13	2.11222676	0.0188254
GOTERM_BP_FAT	GO: 0010558∼negative regulation of macromolecule biosynthetic process	12	2.580653366	0.0063959
GOTERM_BP_FAT	GO: 0031327∼negative regulation of cellular biosynthetic process	12	2.516252034	0.007676
GOTERM_BP_FAT	GO: 0009890∼negative regulation of biosynthetic process	12	2.463555657	0.0089258
GOTERM_BP_FAT	GO: 0006915∼apoptosis	12	2.344879388	0.0126006
GOTERM_BP_FAT	GO: 0012501∼programmed cell death	12	2.310339429	0.0139488
GOTERM_CC_FAT	GO: 0070013∼intracellular organelle lumen	23	1.530125122	0.0368931
GOTERM_CC_FAT	GO: 0043233∼organelle lumen	23	1.495655271	0.0458439
GOTERM_CC_FAT	GO: 0031981∼nuclear lumen	21	1.714061303	0.0165472
GOTERM_CC_FAT	GO: 0016323∼basolateral plasma membrane	6	3.498084291	0.0278083
GOTERM_CC_FAT	GO: 0000123∼histone acetyltransferase complex	5	11.83518519	7.95E-04
GOTERM_CC_FAT	GO: 0016591∼DNA-directed RNA polymerase II, holoenzyme	5	7.305669867	0.0047125
GOTERM_CC_FAT	GO: 0005912∼adherens junction	5	3.817801673	0.0411102
GOTERM_CC_FAT	GO: 0033276∼transcription factor TFTC complex	3	25.36111111	0.0059162
GOTERM_CC_FAT	GO: 0005669∼transcription factor TFIID complex	3	16.13888889	0.0143787
GOTERM_CC_FAT	GO: 0070461∼SAGA-type complex	3	15.43719807	0.015663
GOTERM_MF_FAT	GO: 0003677∼DNA binding	29	1.495570968	0.021546
GOTERM_MF_FAT	GO: 0030528∼transcription regulator activity	22	1.749130413	0.0110444
GOTERM_MF_FAT	GO: 0003700∼transcription factor activity	16	1.972725546	0.0136976
GOTERM_MF_FAT	GO: 0003712∼transcription cofactor activity	8	2.649321498	0.030379
GOTERM_MF_FAT	GO: 0005525∼GTP binding	8	2.58522501	0.0340327
GOTERM_MF_FAT	GO: 0019001∼guanyl nucleotide binding	8	2.517548963	0.0384249
GOTERM_MF_FAT	GO: 0032561∼guanyl ribonucleotide binding	8	2.517548963	0.0384249
GOTERM_MF_FAT	GO: 0016564∼transcription repressor activity	7	2.662945382	0.0464633
GOTERM_MF_FAT	GO: 0003713∼transcription coactivator activity	6	3.370456906	0.0319331
GOTERM_MF_FAT	GO: 0004468∼lysine N-acetyltransferase activity	4	12.6539961	0.0037232
GOTERM_MF_FAT	GO: 0003677∼DNA binding	29	1.495571	0.021546
GOTERM_MF_FAT	GO: 0030528∼transcription regulator activity	22	1.74913	0.011044
GOTERM_MF_FAT	GO: 0003700∼transcription factor activity	16	1.972726	0.013698
GOTERM_MF_FAT	GO: 0003712∼transcription cofactor activity	8	2.649321	0.030379
GOTERM_MF_FAT	GO: 0005525∼GTP binding	8	2.585225	0.034033
GOTERM_MF_FAT	GO: 0019001∼guanyl nucleotide binding	8	2.517549	0.038425
GOTERM_MF_FAT	GO: 0032561∼guanyl ribonucleotide binding	8	2.517549	0.038425
GOTERM_MF_FAT	GO: 0016564∼transcription repressor activity	7	2.662945	0.046463
GOTERM_MF_FAT	GO: 0003713∼transcription coactivator activity	6	3.370457	0.031933
GOTERM_MF_FAT	GO: 0004468∼lysine N-acetyltransferase activity	4	12.654	0.003723
KEGG_PATHWAY	hsa05200: Pathways in cancer	9	2.847498756	0.0107241
KEGG_PATHWAY	hsa04510: Focal adhesion	6	3.097776424	0.0396646
KEGG_PATHWAY	hsa04910: Insulin signaling pathway	5	3.843537415	0.0375672
KEGG_PATHWAY	hsa04210: Apoptosis	5	5.964109782	0.0087712
KEGG_PATHWAY	hsa04130: SNARE interactions in vesicular transport	3	8.192803437	0.049457

DEGs, differential expressed genes.

**Table 2:** Functional analysis of module 1 and module 2.

MCODE	GO term	Description	Log10 (*P* value)
MCODE_1	GO: 1904668	Positive regulation of ubiquitin protein ligase activity	3.267606
MCODE_1	GO: 1901575	Organic substance catabolic process	3.267606
MCODE_1	GO: 0042176	Regulation of protein catabolic process	3.267606
MCODE_2	GO: 0097194	Execution phase of apoptosis	4.399027
MCODE_2	GO: 1901216	Positive regulation of neuron death	4.027797
MCODE_2	GO: 0097202	Activation of cysteine-type endopeptidase activity	4.027797

MCODE, Molecular complex detection; GO, differential expressed genes; KEGG, kyoto encyclopedia of genes and genomes.

**Table 3:** The10 most significant small molecule drugs for Inflammatory breast cancer.

Rank	Cmap name	Mean	*n*	Enrichment score	*P* value
11	Atracurium besilate	−0.648	3	−0.909	0.00132
4	Adiphenine	−0.743	5	−0.894	0.00004
88	Imatinib	−0.571	2	−0.873	0.03187
25	Prestwick-1082	−0.577	3	−0.862	0.00533
6	3-acetamidocoumarin	−0.62	4	−0.86	0.00068
1	Monensin	−0.624	6	−0.859	0.00002
7	Prestwick-692	−0.572	4	−0.855	0.00082
10	Naringenin	−0.647	4	−0.837	0.00125
14	Viomycin	−0.588	4	−0.809	0.00261
159	Arachidonyltrifluoromethane	−0.574	2	−0.809	0.07249

**Table 4:** Relationship between CASP9 expression and clinical parameters of breast cancer patients using the bc-GenExMiner database.

Variables	Patient number	CASP9 mRNA	*P*-value^*∗*^
ER			<0.0001
Negative	7038		
Positive	2550	Increased	

PR			<0.0001
Negative	3377		
Positive	2597	Increased	

HER-2			<0.0001
Negative	4581	Increased	
Positive	778		

P53 status			<0.0001
Wild type	1328	Increased	
Mutated	652		

Basal-like status			<0.0001
Non-basal-like	7990	Increased	
Basal-like	2075		

Triple-negative status			<0.0001
Non-triple-negative	7566	Increased	
Triple-negative	897		

**Table 5:** CASP9 expression and survival data of breast cancer patients using the PrognoScan database.

Dataset	Endpoint	Probe ID	N	Minimum *P*-value	HR [95% CI-low CI-upp]
GSE4922-GPL96	Disease free survival	203984_s_at	249	0.000875239	0.48 [0.22–1.05]
GSE7378	Disease free survival	203984_s_at	54	0.00780304	3.03 [0.70–13.12]
GSE4922-GPL96	Disease free survival	210775_x_at	249	0.000545375	0.18 [0.06–0.58]
GSE7378	Disease free survival	210775_x_at	54	0.0349634	2.54 [0.68–9.46]
GSE7849	Disease free survival	486_at	76	0.00164014	1.88 [0.79–4.46]
E-TABM-158	Disease specific survival	203984_s_at	117	0.0128229	2.28 [1.15–4.51]
GSE3494-GPL96	Disease specific survival	203984_s_at	236	0.00956079	0.42 [0.15–1.17]
E-TABM-158	Disease specific survival	210775_x_at	117	0.0119705	2.13 [0.95–4.80]
GSE1456-GPL96	Disease specific survival	210775_x_at	159	0.0245736	3.22 [0.48–21.52]
GSE3494-GPL96	Disease specific survival	210775_x_at	236	0.00981181	0.28 [0.06–1.25]
E-TABM-158	Distant metastasis free survival	203984_s_at	117	0.0178118	2.59 [1.18–5.65]
GSE11121	Distant metastasis free survival	203984_s_at	200	0.000741152	0.31 [0.10–0.93]
GSE19615	Distant metastasis free survival	203984_s_at	115	0.0309658	0.26 [0.03–2.41]
GSE2034	Distant metastasis free survival	203984_s_at	286	0.000828995	0.48 [0.23–1.00]
GSE2990	Distant metastasis free survival	203984_s_at	54	0.000491493	0.66 [0.33–1.31]
GSE6532-GPL570	Distant metastasis free survival	203984_s_at	87	0.014011	0.40 [0.15–1.06]
GSE7390	Distant metastasis free survival	203984_s_at	198	0.0145058	0.55 [0.29–1.05]
GSE11121	Distant metastasis free survival	210775_x_at	200	0.000167631	0.15 [0.05–0.42]
GSE19615	Distant metastasis free survival	210775_x_at	115	0.00314842	0.13 [0.01–1.47]
GSE2034	Distant metastasis free survival	210775_x_at	286	0.0127349	0.48 [0.26–0.90]
GSE2990	Distant metastasis free survival	210775_x_at	54	5.82E-05	0.81 [0.50–1.33]
GSE6532-GPL570	Distant metastasis free survival	210775_x_at	87	0.00341454	0.51 [0.18–1.42]
GSE7390	Distant metastasis free survival	210775_x_at	198	0.0375517	0.66 [0.31–1.39]
GSE9195	Distant metastasis free survival	210775_x_at	77	0.0185039	0.48 [0.07–3.48]
GSE9893	Overall survival	21619	155	0.000532692	1.53 [1.15–2.03]
E-TABM-158	Overall survival	203984_s_at	117	0.014406	2.15 [1.19–3.87]
GSE7390	Overall survival	203984_s_at	198	0.0134952	0.51 [0.26–1.00]
E-TABM-158	Overall survival	210775_x_at	117	0.008304	2.12 [1.06–4.26]
GSE7390	Overall survival	210775_x_at	198	0.0195291	0.52 [0.24–1.12]
GSE3143	Overall survival	486_at	158	0.0109667	1.22 [0.80–1.85]
GSE3143	Overall survival	487_g_at	158	0.0123634	0.66 [0.33–1.30]
GSE1379	Relapse free survival	13400	60	0.0229978	0.51 [0.20–1.29]
E-TABM-158	Relapse free survival	203984_s_at	117	0.014406	2.15 [1.19–3.87]
GSE12276	Relapse free survival	203984_s_at	204	0.0371474	0.79 [0.47–1.32]
GSE1456-GPL96	Relapse free survival	203984_s_at	159	0.0387143	0.78 [0.29–2.10]
GSE2990	Relapse free survival	203984_s_at	62	0.00529065	0.72 [0.41–1.28]
GSE2990	Relapse free survival	203984_s_at	125	0.00603378	0.48 [0.18–1.29]
GSE6532-GPL570	Relapse free survival	203984_s_at	87	0.014011	0.40 [0.15–1.06]
GSE7390	Relapse free survival	203984_s_at	198	0.0305851	0.87 [0.52–1.47]
GSE9195	Relapse free survival	203984_s_at	77	0.0195901	0.38 [0.08–1.86]
E-TABM-158	Relapse free survival	210775_x_at	117	0.008304	2.12 [1.06–4.26]
GSE1456-GPL96	Relapse free survival	210775_x_at	159	0.00868802	3.13 [0.65–15.01]
GSE2990	Relapse free survival	210775_x_at	62	0.00589812	0.84 [0.56–1.26]
GSE6532-GPL570	Relapse free survival	210775_x_at	87	0.00341454	0.51 [0.18–1.42]
GSE9195	Relapse free survival	210775_x_at	77	0.0189982	0.44 [0.08–2.47]

## Data Availability

The data used to support the findings of this study are included within the article.

## Supplementary Materials

Supplementary Materials Graphical Abstract Text. The Comparative Toxicogenomics Database (CTD) was applied to analyze the literature of the candidate compounds and gene. Through the gene-compound interaction network, CASP9-NGE is expected to be the most promising candidate gene-compound interaction for the treatment of IBC.
