# Supplementary figures and images for: CASP9 As a Prognostic Biomarker and Promising Drug Target Plays a Pivotal Role in Inflammatory Breast Cancer
Source: Int J Anal Chem. 2022 Sep 25;2022:1043445. doi: 10.1155/2022/1043445 (PMC9527435; doi:10.1155/2022/1043445)

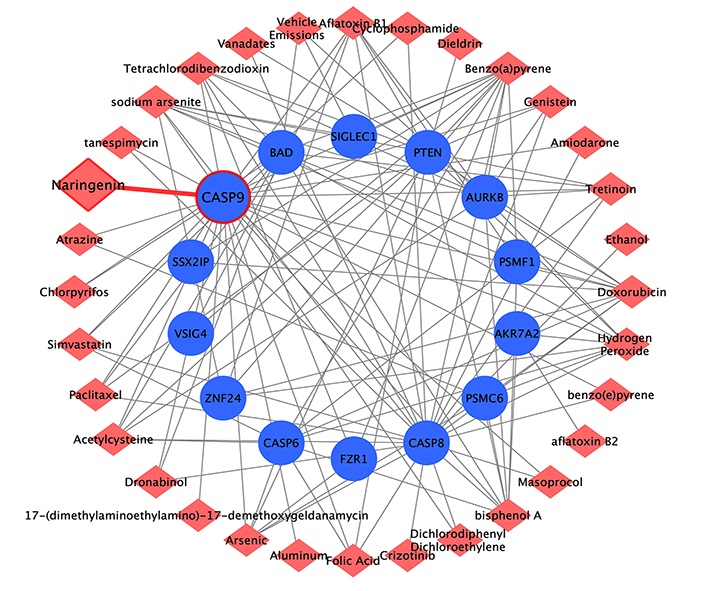

Supplement: Supplementary Materials — Graphical Abstract Text. The Comparative Toxicogenomics Database (CTD) was applied to analyze the literature of the candidate compounds and gene. Through the gene-compound interaction network, CASP9-NGE is expected to be the most promising candidate gene-compound interaction for the treatment of IBC. [file 1043445.f1.jpg]
